# The role of midwives in supporting the development of the mother-infant relationship: a scoping review

**DOI:** 10.1186/s40359-023-01092-8

**Published:** 2023-03-14

**Authors:** Cathy Stoodley, Lois McKellar, Tahereh Ziaian, Mary Steen, Jennifer Fereday, Ian Gwilt

**Affiliations:** 1 University of South Australia, South Australia, Australia; 2 School of Health and Social Care, Edinburgh Napier University, Edinburgh, Scotland; 3 University of Northumbria, Newcastle, England

**Keywords:** Mother-Child Relations, Object attachment, Midwifery, Health promotion, Midwife

## Abstract

**Background:**

The mother-infant relationship is complex and dynamic, informing the psychological development of the infant through bonding and attachment. Positive early interactions influence the quality of this relationship. Midwives are well placed to support the developing relationship between the mother and baby, yet there has been limited research exploring the role of the midwife in this context.

**Aim:**

To explore interventions that have been provided by the midwife which support the development of the maternal-fetal or mother-infant relationship amongst a low-risk population from pregnancy, and up to six weeks postnatal. The review also sought to understand the types of interventions developed, format and delivery, outcomes measured and if cultural considerations had been incorporated.

**Methods:**

A scoping review of the research literature was undertaken using the Joanna Briggs Institute framework. Five online databases were searched for relevant articles published in English from 2000 to 2021.

**Findings:**

Sixteen articles met the inclusion criteria. Three themes emerged: (1) viewing the fetus as separate from the mother, (2) focused activities on the maternal-infant relationship and (3) targeted educational interventions.

**Discussion:**

Providing focused activities and targeted education during the pre and postnatal periods support the development of the mother-infant relationship. Significantly, there was insufficient research that considered the influence of culture in supporting the mother-infant relationship.

**Conclusion:**

Further research is required to develop interventions that include a diverse sample to ensure culturally appropriate activities can be integrated into care during pregnancy and/or the postnatal period provided by midwives.

**Supplementary Information:**

The online version contains supplementary material available at 10.1186/s40359-023-01092-8.

## Introduction

The mother-infant relationship is complex and dynamic, influencing both the mother and her infant, with the relationship developed through an interactional partnership involving a variety of physical, cognitive, social and affective behaviours [1]. Positive early interactions can influence the quality of the mother-infant relationship and are essential for the healthy psychological development of the infant [2].

### Mother-infant relationship

The mother-infant relationship is commonly discussed in terms of bonding and attachment. Maternal-infant bonding is described as a one-way connection from the mother to the infant, involving maternal emotions and feelings [3–5]. Whereas maternal-infant attachment is described as the reciprocal relationship between the mother and their infant, which is characterised by how the child uses their caregiver as a secure base during exploration [6].

This bond between the mother and infant develops progressively during pregnancy (*known as the maternal-fetal bond*), after birth and until early childhood [3–5]. During pregnancy, the maternal-fetal bond develops through activities where the mother communicates with the unborn baby, engages with fantasies, has mental images and develops future plans with the baby [7]. The quality of the maternal-fetal bond plays an important role in the mother-infant relationship and can be an indicator of postnatal maternal sensitivity and the mother-infant bond [8]. In the first few hours after birth, the mother-infant bond is influenced by hormones that enhance maternal sensitivity, reactivity, and receptiveness to their baby [4]. In addition to maternal hormones, changes in neurochemistry also occur, which increases the plasticity of the maternal brain, promoting maternal caregiving behaviours [9].

In the early postnatal period, the maternal-infant relationship continues to be influenced by close continual physical contact and maternal interactions with their infant. For the healthy development of the maternal-infant bond, it is important that the mother regularly engages in nurturing activities such as cuddling, rocking, singing, feeding, gazing, and kissing [10]. It is also essential that there is face-to-face interaction, eye contact, skin-to-skin, touching, smelling, and smiling [10, 11]. A high-quality maternal-infant bond occurs when the mother can provide warm, close, continuous, affectionate behaviour towards her infant and where comfort and pleasure for both the mother and the infant take place [12]. The quality of this bond can be influenced by both maternal and infant factors [4]. Poorer quality bonding is associated with a lack of positive maternal feelings, irritability, hostility and possible rejection of the infant [3]. These first experiences between mother and baby are crucial to forming the infant’s foundation for relationships across their lifetime [12], with the mother-infant bond a precursor for the development of secure attachment [13].

Numerous studies have investigated the mother-infant relationship identifying factors including breastfeeding [14], maternal mental health [15], perinatal depression [16, 17], preterm birth [18], infants with colic, infants with or at risk of developing a sleeping problem, and parents with an insensitive parenting style [19] that may negatively influence the developing relationship. In response, interventions have been developed, largely responding to the impact that a disrupted mother-infant relationship can have on infant wellbeing. Most commonly these interventions are delivered by psychologists, nurses and allied health clinicians, with limited research exploring midwifery-led interventions during pregnancy or in the early postnatal period.

Notably, studies on the maternal-infant relationship have primarily focused on Western middle-class Caucasian populations [20], with limited research which considers the influence of culture on the mother-infant relationship [21]. However, there is agreement that irrespective of culture, all infants require warm, responsive, protective and linguistically rich environments from their primary caregiver for appropriate growth and development.

### Role of the midwife

In the late 70s, Kennell and Klaus conceptualised the ‘sensitive’ period as the first few hours or days after birth and emphasized the importance of supporting positive mother-infant contact to enhance the developing relationship. Since then, research has redefined the sensitive period to include pregnancy through to early childhood [22]. Midwives are ideally placed to support women in developing the emerging relationship with their infant as their scope of practice commences with pregnancy and concludes between six to eight weeks postnatal [23]. Midwifery care involves the midwife and woman working in partnership together, promoting healthy pregnancy, physiological birth and a positive transition to motherhood. Initiatives such as ‘rooming in’ to nurture the mother-infant dyad and the Baby-Friendly Hospital Initiative (BFHI) [now known as Baby-Friendly Health Initiative] introduced in 1991, are examples of strategies that the midwife has promoted to enhance the well-being of mothers and their babies. Further research is warranted to identify ways the midwife can support mothers in the early mother-infant relationship.

This paper reports the findings of a scoping review that initially aimed to identify interventions provided by midwives to support the development of the maternal-fetal or mother-infant relationship in a low-risk population of women. Recognising that globally, midwifery care is provided within varied contexts and across the disciplines of both nursing and midwifery, the scoping review was extended to include nurses working in a maternity setting, as there was very limited research that reported on midwives distinctly. Additionally, the review sought to explore the types of interventions developed, including the format and delivery, outcome measurements and if any cultural considerations had been included.

## Methodology and methods

The scoping review was conducted following the JBI nine-step process by Peters and colleagues [24] (see Table 1). It also utilised the Preferred Reporting Items for Systematic reviews and Meta-Analyses extension for Scoping Reviews (PRISMA-ScR) guidelines [25]. A scoping review was undertaken to systematically map the research and included identifying any existing gaps in knowledge [26, 27].

**Table 1:** JBI Scoping Review Framework developed by Peters et al. [24]

Step 1. Defining and aligning the objective/s and question/s
Step 2. Developing and aligning the inclusion criteria with the objective/s and question/s
Step 3. Describing the planned approach to evidence searching, selection, data extraction, and presentation of the evidence
Step 4. Searching for the evidence
Step 5. Selecting the evidence
Step 6. Extracting the evidence
Step 7. Analysis of the evidence
Step 8. Presentation of the results
Step 9. Summarizing the evidence in relation to the purpose of the review, making conclusions and noting any implications of the findings

Adapted from: JBI 2020, Scoping Review [24]

### Defining the objectives and question

This scoping review aimed to identify and explore interventions that have been provided by the midwife and/or nurse which support the development of the maternal-fetal or mother-infant relationship amongst a low-risk population from pregnancy, and up to six weeks postnatal.

Objectives.

i. what types of interventions have been developed?
ii. what type of format and delivery options has occurred?
iii. were any outcomes measured?
iv. has any cultural inclusion been considered?

### Developing the inclusion criteria

**Fig. 1:**
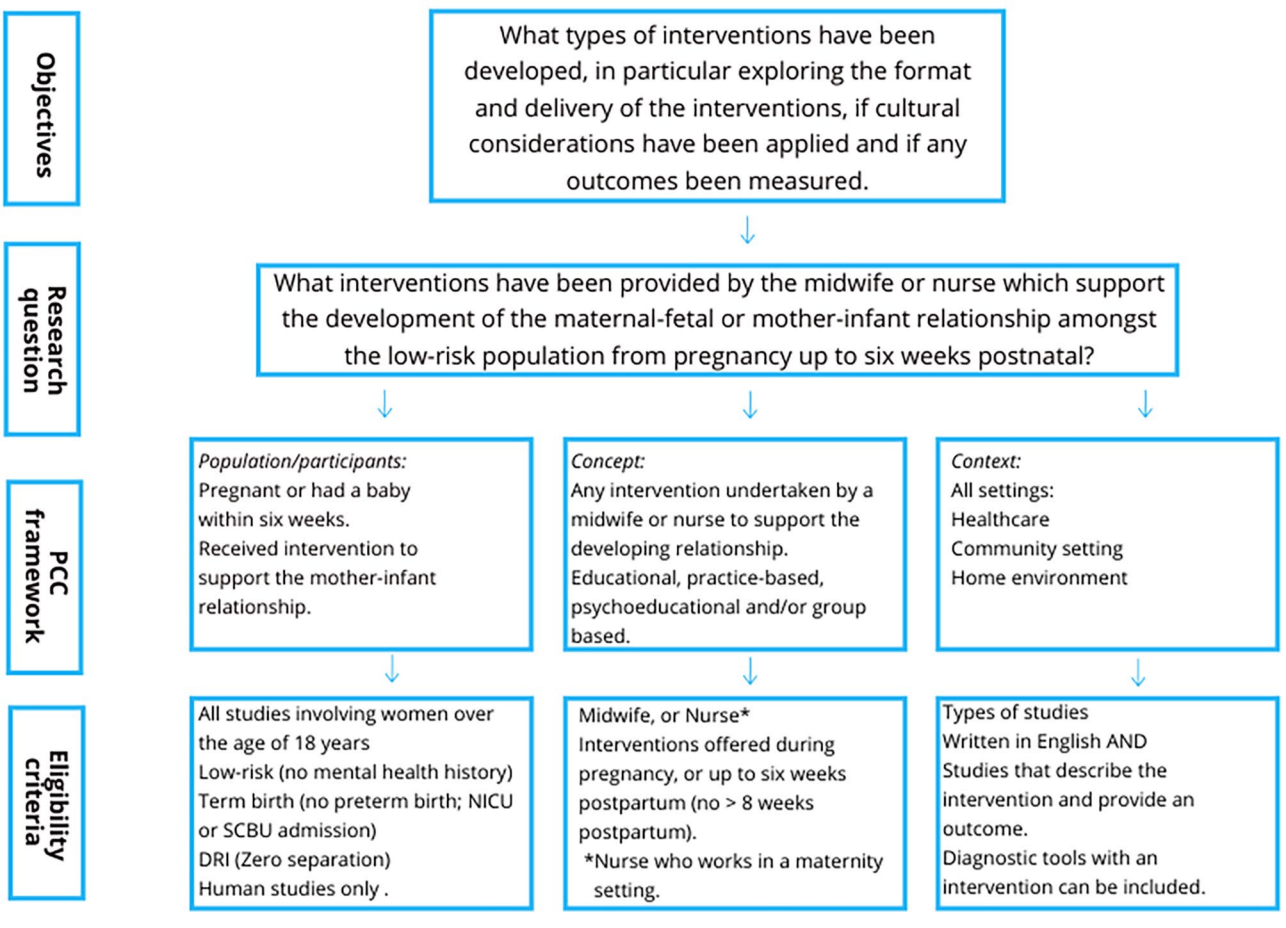
Relationship between research objectives, questions and eligibility criteria [24]

The PCC (*Participants, Concept and Context*) framework was utilised in the process of developing the research question, objectives, inclusion and exclusion criteria and also the literature search strategy. The eligibility criteria are directly linked to the research objectives and question [26, 27] (see Fig. 1).

### The planned approach to finding evidence

A *prior protocol* (available from the open science framework) was developed in consultation with an Academic Librarian (LD) who assisted to define the search strategy and identify the relevant databases [26]. The search strategy, including all identified keywords and index terms, was adapted for each database. Search techniques included using medical subject headings (MeSH) and Boolean operators to widen, narrow, or combine search results. The reference list of all studies was screened for additional studies. Only published studies in the English language were included, from 2000 to 2021 to reflect contemporary midwifery practice. It was necessary to apply an exclusion criterion of animal studies and the non-infant (child, adolescent, or adult) population. When full-text articles were either not available or not published in the English language the authors were contacted via email to try and obtain the article.

### Searching for the evidence

In collaboration with an academic librarian (LD), the evidence search was undertaken in three stages. Stage one involved an initial limited search of MEDLINE (Ovid) and PsycInfo to identify articles on the topic. The text words contained in the titles and abstracts of relevant articles, and the index terms used to describe the articles were used to develop a full search strategy (see supplementary 1 for full search strategy). In order to capture the breadth of the mother-baby relationship numerous key search terms were needed and included: Mother-infant, mother-child, maternal-infant, maternal-foetal, pregnancy, fetus, infant, newborn, postpartum, attachment, bond, nurse, midwife, nurse-midwife, program, intervention, education, health promotion. Stage two involved searching for evidence across a broad range of relevant electronic databases such as Ovid Medline, Ovid Embase, Ovid Emcare, PsycINFO and Scopus using the same search strategy. Then (CS) conducted a hand search of the reference lists from the included studies.

### Selecting the evidence

The studies were retrieved from databases, imported into Endnote X9 (Clarivate Analytics, PA, USA), and then into Covidence, (Melbourne, Australia) for screening, where duplicates were removed. The eligibility criteria (see Fig. 1) informed the study selection, with the management of disagreements undertaken in conjunction with three authors (LM, TZ, MS). The approach used to pilot the source selection involved developing the inclusion and exclusion criteria undertaken by (CS, LM) (see Table 2). An initial review of the sample [15] was undertaken by (CS) and then discussed with (LM). Following the pilot test, the titles and abstracts were screened by the lead author (CS), and then the full text of selected citations was assessed by two independent reviewers (CS, LM). Any disagreements between the two reviewers (CS, LM) were managed by consensus. The reasons for exclusion at full text have been identified in the PRISMA flowchart of articles screened for inclusion in the scoping review.

**Table 2:** Inclusion and exclusion criteria

Inclusion criteria	Exclusion
Over 18 years of age An intervention provided by a midwife/maternity nurse/nurse Supporting mother-infant relationship Low-risk (no mental health history) Term birth Interventions offered during pregnancy, postnatal to eight weeks (maternal-fetal & mother-infant relationship) Direct Room In (Zero separation) Screening or assessment tools with an intervention Written in English	Under 18 years of age An intervention provided by other health-professional Pharmacological intervention The population includes women with mental illness Reported substance abuse (smoking, alcohol, recreational) Interventions that target high-risk mother-infant relationship disturbances Interventions offered after 8 weeks of age No reported evaluation Special Care Nursery (SCN) or Neonatal Intensive care (NICU) interventions Preterm birth Screening or assessment with no intervention Not written in English

### Extracting the evidence

A standardized data extraction form was selected during the development of the protocol and initially piloted with several papers in consultation with the reviewer (LM) [26]. The final data extraction form included: the article title, authors, journal, date of publication, population, context, concept, methodology, outcomes and key findings. A critical appraisal and risk of bias assessment of the studies were not conducted during the review as the aim of this scoping review was to map the available evidence not to provide a synthesized answer to a question [27].

### Analysis of the evidence

**Fig. 2:**
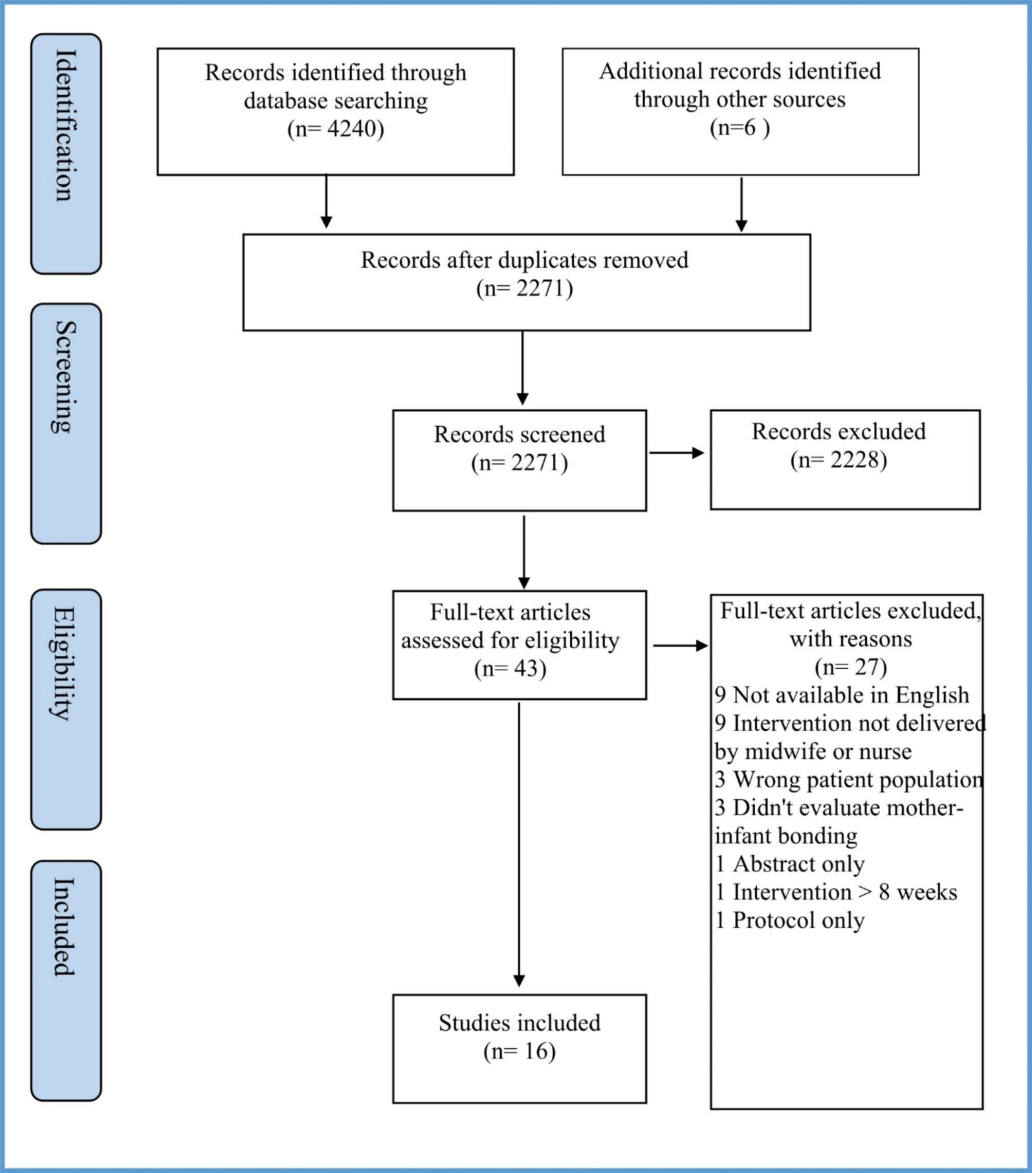
PRISMA flowchart of articles screened for inclusion in the scoping review [25]

A total of 2271 studies were reviewed, of these, 43 full-text studies were assessed for eligibility. Sixteen studies met the inclusion criteria and were included in this review. The study selection process was conducted following the PRISMA-ScR guidelines [28] (see Fig. 2).

## Results

### Characteristics of the included studies

There were a variety of countries represented in the review, with three undertaken in Turkey [29–31] and Norway [32–34], and two studies in Italy [35, 36]. One study in Japan [37], Sweden [38], Korea [39], Brazil [40], the United States of America [41], Switzerland [42], Colombia [43], and Taiwan [44]. The variety of study designs used is outlined in Table 3.

**Table 3:** Types of studies in this scoping review

Quantitative	• 3 X Randomised Control Trial [RCT] (29, 37, 43) • 3 X Quasi-randomised design (30, 35, 36) • 1 X Quasi-experimental pattern with a control group on pre-test and post-test design (31) • 1 X Longitudinal study, with a non-randomised cluster-controlled design (32) • 1 X Prospective population-based survey (38) • 1 X Pre-experimental design with pre and post-test (39) • 1 X Single-blind multiple time series design (44) • 1 X Pilot study with, pre and post-comparison design (42) • 1 X Cross-sectional study (40) • 1 X Feasibility study (41)
Qualitative	• 1 X Explorative design (33) • 1 X Phenomenology (34)

Interventions that were provided by a midwife and/or nurse could be described within three themes: [1] viewing the fetus as separate from the mother, [2] focused activities on the maternal-infant relationship and [3] targeted educational interventions.

### Viewing the fetus as separate from the mother

Three studies [29, 37, 38] reported activities that promoted maternal-fetal interaction during pregnancy. These activities involved the midwife supporting the woman to develop an awareness of the fetus and included abdominal palpation and perception of fetal movements. Nishikawa and Sakakibara [37] proposed that an intervention program involving abdominal palpation may support the development of maternal-fetal attachment. Similarly, Celik and Ergin [29] used an abdominal palpation intervention to explore maternal-fetal attachment. The authors proposed that midwives/nurses may be able to promote practices that improve the woman’s awareness of the fetus during their pregnancy, in turn strengthening the prenatal attachment. Whereas Malm and colleagues [38], specifically explored fetal movements over 24 h to investigate the impact on maternal-fetal attachment. The authors proposed that a positive prenatal attachment in late pregnancy may be experienced by women who have a greater perception of fetal movements over 24 h.

The format and delivery of the interventions varied (see Table 4), with two studies [29, 37] involving education strategies, with the midwife utilising both teaching and demonstration techniques. One study did not provide education but sought to understand maternal perceptions through questionnaires [38]. All three studies measured maternal-fetal attachment using self-reported validated tools. These studies did not specifically consider cultural factors, although Celik and Ergin [29] recommended that culturally specific studies should be conducted.

**Table 4:** Study format, delivery, tools and cultural inclusion

Article	Format	Delivery of sessions	Validated tools to measure bonding or attachment	Was there any cultural inclusion
Celik and Ergin (29)	The participants received education on embryo-fetal development, feedback on the antenatal palpation assessment and demonstration of the first and second Leopold’s manoeuvre. The women were invited to complete the maneuvers and listen to the fetal heartbeat on themselves.	Sessions occurred in the third trimester of pregnancy. Number of sessions offered: 2 (*32nd and 36th gestational weeks*) Length of intervention: 45 to 60 min Group sessions: Maximum of five participants per session.	The Prenatal Attachment Inventory (PAI) was developed by Muller (45).	No mention in the paper of any cultural factors included. The authors did note in the conclusion and recommendations that culturally specific studies should be conducted.
Malm et al. (38)	As this study was a prospective population-based survey no education was provided. The authors sought to understand maternal perception by using questionnaires.	Women in the third trimester were invited to complete the questionnaires.	Prenatal Attachment Inventory–Revised (PAI-R) developed by Pallant and colleagues (46).	Swedish women were recruited. No mention in the paper of any cultural factors included.
Nishikawa and Sakakibara (37)	The participants were provided with an explanation of fetal positions and invited to touch the buttocks, back and small and larger body parts of the fetus during an abdominal examination. This was followed by a group discussion.	Sessions occurred in the third trimester of pregnancy The number of sessions offered: 3 (*30th, 32nd, and 34th weeks gestation*). Length of intervention: 1 to 1.5 h. Group discussion	PAI Japanese version of the by Tujino and colleagues (47).	Japanese women were recruited. No mention in the paper of any cultural factors included.

The outcomes reported for the abdominal palpation interventions demonstrated that the PAI score in the intervention group was significantly higher (P < .01) at the 32nd, 34th, and 36th weeks gestation compared to the baseline [37]. Similarly, Celik and Ergin [29] identified a statistically significant difference between the intervention and control groups, with the women in the intervention group recording a (p < .01) positive effect on their prenatal attachment scores at the 32nd and 36th week of pregnancy. Malm and colleagues [38] exploring fetal movement perception reported that the PAI-R indicated significantly higher scores of attachment for the women who perceived frequent fetal movements on several occasions within 24 h. Higher scores in all subscales on ‘anticipation’, ‘differentiation’ and ‘interaction’ occurred when women perceived many fetal movements on numerous occasions. The common consensus was that interventions that increase maternal awareness through either abdominal palpation or perception of fetal movement, appear to be effective for promoting maternal-fetal attachment.

### Focused activities can enhance the maternal-infant relationship

Strengthening maternal-fetal attachment or mother-infant bonding using focused activities was discussed in six studies. There appeared to be a variety of activities delivered by the midwife or nurse which aimed to promote attachment or bonding undertaken in either the prenatal [35, 39], prenatal and postnatal period [36] or the postnatal period [30, 33, 40].

Some of these focused activities occurred only during pregnancy, with Bellieni and colleagues [35] providing a prenatal education course (PEC) that focused on communication between the mother and fetus to support both prenatal and postnatal attachment. Chang, Park and Chung [39] also utilised the antenatal period to conduct an educational course that proposed that integrating western ideas with Korean traditional practices could positively impact maternal-fetal attachment. Similarly, Persico and colleagues [36] also commenced their study during the prenatal period however, they investigated the impact of maternal singing on bonding, newborn behaviour and maternal stress. The authors proposed a lullaby singing intervention, where the mother sang traditional, loving and playful songs to enhance the prenatal attachment and postnatal mother-infant bonding.

There were also three studies [30, 33, 40] conducted in the postnatal period. Gürol and Polat [30] investigated if the attachment between the mother and baby is strengthened by performing infant massage. Likewise, Midtsund, Litland, and Hjalmhult [33] investigated the effects of infant massage, specifically exploring the experiences of the mothers participating in the Mamma Mia programme. While Helk Souza and colleagues [40] explored whether mother-infant bonding was influenced by the experiences women had during and after labour.

The format and delivery of the interventions varied and primarily involved focused activities (see Table 5). One study did not provide education but sought to understand the influence that specific experiences may have on bonding through a questionnaire [40]. Four studies [30, 35, 36, 39] measured either maternal-fetal or mother-infant bonding or attachment using self-reported validated tools. One study was qualitative therefore no tool was used [33]. Interestingly, Persico and colleagues [36] were the only study to explore attachment and bonding in both the prenatal and postnatal periods. Only one study specifically explored bonding from a cultural perspective, integrating western ideas with Korean traditional practices [39]. Although Persico and Colleagues [36] acknowledged that lullaby singing is a part of traditions and cultures.

**Table 5:** Study format, delivery, tools and cultural inclusion

Article	Format	Delivery of sessions	Validated tools to measure bonding or attachment	Was there any cultural inclusion
Bellieni and colleagues (35)	The PEC design was influenced by Veldman’s (48) studies of haptonomy. This approach involved raising maternal awareness of fetal presence and development while learning how to interact with the fetus, through gentle stimulation and perceiving the responses. The prenatal sessions varied, with a combination of prenatal education and activities which included the basics of fetal physiology and development, singing, dancing and massage-through-the-womb sessions	Sessions occurred during pregnancy. The authors did not provide any information on where the sessions took place Number of sessions offered: 5 Length of session:1 h Group sessions	The Prenatal Attachment Inventory (PAI) was developed by Muller (46).	No mention in the paper of any cultural factors included.
Chang, Park and Chung (39)	The sessions used a combination of approaches and drew on the Taegyo-focused program developed by Chang and colleagues (45) involving lectures, demonstrations, practice, training, discussion, and sharing of personal experiences. The prenatal sessions included standard prenatal class information but added information specific to Taegyo practices, including understanding the ability of the fetus to respond, sharing motivations, the purpose of pregnancy, preconceptions of experiencing childbirth, training in maternal-fetal interaction, writing letters and making a declaration of love to the unborn baby.	Sessions occurred during pregnancy (*20 to 36 gestational weeks*). The sessions were offered in health education rooms in a public health centre Number of sessions offered: 4 weeks Length of session:2 h Group sessions	The Maternal-Fetal Attachment Scale (MFAS) was developed by Cranley (50).	Yes, Taegyo-focused prenatal classes.
Gürol and Polat (30)	The researchers provided educational sessions and a demonstration on how to perform baby massage which was supplemented with educational brochures and compact disks (CDs). Mothers then performed a daily massage on their babies for the period of the study.	The session was conducted in the participant’s home week after the birth (*between 5 to 7 days postnatal*). The number of sessions offered: daily for 38 days. Length of session: 15 min. One to one	Maternal Attachment Inventory (MAI) developed by Müller (52)	No mention in the paper of any cultural factors included.
Helk Souza and colleagues (40)	No specific educational intervention was implemented, and data was collected on the type of birth, pain during birth, and skin-to-skin contact due to the influence that these experiences may have on the maternal-infant bond.	None	Mother-To-Infant Bonding Scale (MIBS) developed by Taylor et al. (51).	No mention in the paper of any cultural factors included.
Midtsund, Litland, and Hjalmhult (33)	The Mamma Mia programme taught mothers how to perform an infant massage to improve interactions with their babies. It involved two aspects, the first was focused on teaching mothers about infant massage and the second was teaching about topics such as interaction, attachment, and children’s behaviour.	Sessions were conducted in health service centres such as the Well Child Clinic (WCC). Babies from 1-month-old. Number of sessions offered: weekly for 6 weeks Length of session: not described. Group setting	As this was a qualitative study there were no tools used.	No mention in the paper of any cultural factors included.
Persico and colleagues (36)	Before the participant’s first antenatal class, they were provided nine lullabies and practised each lullaby with the midwife. After completing this task for four weeks participants were invited to choose two lullabies and encouraged to continue singing at home. The participants were asked to record the frequency of singing each week, their emotions, the response of the fetus, and after birth, to observe the baby’s behaviour. The participants also received education about maternal singing during the antenatal classes.	Sessions commenced during pregnancy (from 24 weeks gestation) and were offered in a metropolitan maternity hospital before antenatal classes. Number offered: 14 weekly sessions. Did not describe the length of the sessions. Group sessions	The Prenatal Attachment Inventory (PAI) was developed by Muller (46). Mother-To-Infant Bonding Scale (MIBS) developed by Taylor et al. (51).	No mention in the paper of any cultural factors included. The authors did acknowledge that singing lullabies are part of traditions and cultures all around the world.

The outcomes from the focused activities of prenatal education appear to support the development of prenatal attachment, with Bellieni and colleagues [35] reporting that the PAI score in the intervention group was significantly higher than the control group. These researchers suggested that prenatal education courses which are centred on communication between the mother and fetus may enhance prenatal attachment. Similarly, Chang, Park and Chung [39] reported improved prenatal attachment, with significantly higher pre to post-test (p < .001) from the MFAS score, indicating that the Taegyo-focused prenatal classes positively impacted maternal-fetal attachment scores, specifically, subscales ‘attributing characteristics’ and ‘intentions to the fetus’ and ‘role taking’. Interestingly, Persico and colleagues [36] reported that there was no significant influence on prenatal attachment with maternal singing during pregnancy. Although, the mother-infant bonding score at three months postnatal did demonstrate a statistically significant difference in bonding in the singing group. The study also found that mothers mostly chose to sing in the evening and experienced positive feelings when singing lullabies such as serenity, relaxation and harmony with the baby, along with an increased awareness of changes in fetal behaviour. Most mothers in the singing group continued singing lullabies to their babies after birth and found that this activity enriched their relationship.

Infant massage also appears to be positively associated with supporting mother-infant attachment. Gürol and Polat [30] reported that the MAI scores from the intervention group were significantly higher (p < .001) post-test. The control group reported an increase in maternal attachment score between pre and post-test, however, the score was considerably lower than the intervention group. Similarly, Midtsund, Litland, and Hjalmhult [33] found that infant massage enhanced the relationship, particularly for mothers who struggle with the transition to motherhood. They reported that it was a positive experience and helped them feel attached to their baby during the infant massage and found that they integrated this knowledge into their everyday life. Additionally, infant massage helped establish a deeper connection with their baby and offered an opportunity to spend time with them. Infant massage also provided the mothers with a useful technique to calm their baby, without breastfeeding, with mothers reporting feeling empowered to use what they had learnt. The mothers valued the education that they received on baby cues, signs and behaviours [33]. Helk Souza and colleagues [40] reported that the maternal-infant bond was not significantly influenced by the type of birth or pain experienced during birth. However, it was reported that a lack of early skin-to-skin contact between mother and baby negatively influenced bonding. The mothers who did not have skin-to-skin contact with their newborns showed significantly more sadness than those who did.

### Targeted educational interventions

There were a variety of educational interventions used to support the mother-infant relationship. There were four studies [31, 42–44] that focused on parent education that explored general pre or postnatal topics or psychoeducation, as well as three studies [32, 34, 41] that provided targeted education that focused on enhancing the mother-infant relationship.

Üstüner and colleagues [31] proposed that education and support provided by midwives and/or nurses during pregnancy and the early transition to parenthood through follow-up clinics and health counselling would positively affect the maternal attachment level. Razurel and colleagues [42] explored the impact of standardized pre, and postnatal psycho-educational interviews (PEPI) on mental wellbeing, self-efficacy, the couple’s relationship and the mother-infant relationship. The authors proposed that interventions undertaken in the pre or postnatal period that improve the parental transition can support mother-infant bonding and positively impact the mother-infant relationship.

Vargas-Porras and colleagues [43] explored the efficacy of a multimodal nursing intervention for first-time mothers to support the process of becoming a mother. Similarly, Chung and colleagues [44] explored the quality of the mother-infant interaction and sense of parenting competence for first-time mothers receiving postnatal parenting education.

The Newborn Behavioral Observations (NBO) were utilised in three studies [32, 34, 41], which were delivered by nurses. The NBO aims to sensitise parents to the infant’s capacities, uniqueness, and behavioural communication cues, which can contribute to increased maternal confidence and competence and a positive parent-infant relationship [45]. Sanders and Buckner [41] investigated the NBO as an intervention for first-time mothers to enhance their engagement by increasing their understanding of their infant’s behavioural cues. Similarly, Hoifodt and colleagues [32] evaluated the effectiveness of using the NBO as a universal preventive intervention at a well-baby clinic. While Cheetham and Hanssen [34], specifically sought to understand the impact that the NBO had on the mother-infant relationship, from the mother’s perspective.

The format and delivery of the interventions varied and also primarily involved educational strategies (see Table 6). Four of these studies [31, 32, 42, 43] evaluated attachment or bonding using self-reported validated tools, with Sanders and Buckner [41] using the NBO Parent Questionnaire. One study [34] was qualitative therefore no tool was used to measure bonding or attachment. Notably, Üstüner and colleagues [31] were the only study to explore attachment in both the prenatal and postnatal periods. These studies did not specifically consider cultural factors. Although, Üstüner and colleagues [31] and Sanders and Buckner [41] highlighted that there should be further studies involving different cultures. While Chung and colleagues [44] reported that their findings may have been influenced by Taiwanese cultural factors such as modesty and humility.

**Table 6:** Study format, delivery, tools and cultural inclusion

Article	Format	Delivery of sessions	Validated tools to measure bonding or attachment	Was there any cultural inclusion
Üstüner and colleagues (31)	Education provided on topics such as pregnancy and well-being, physiology of birth and techniques to cope with birth waves (contractions), Pilates exercises, hospital bag preparation, importance of breast milk and breastfeeding techniques, newborn care and common problems, nursing care and family planning methods and a preview of the birthing room.	Sessions occurred during pregnancy (*20 and 30 weeks gestation*). The sessions took place in a maternity clinic in the hospital. Number of sessions offered: 4 Length of session: Not described Group sessions	Used the Turkish version of The prenatal Attachment Scale developed by Yılmaz & Beji (57) and Maternal Attachment Scale developed by Kavlak & Sirin (58).	No mention in the paper of any cultural factors included. In the discussion, the authors did highlight that there should be further studies involving different cultures.
Razurel and colleagues (42)	There were four stages involved in the PEPI which aimed to decrease perceptions of adaptation-associated stress by assisting mothers to find solutions that suit their individual needs and promoting the development of personal resources while increasing the sense of self-efficiency.	The sessions took place in a community setting. The pregnancy interview occurred at 30 weeks gestation and postnatal interview at 6 weeks after birth. Number of sessions offered: 2 Length of session: 1 h One-to-one sessions	Maternal Attachment Scale (MAS).	No mention in the paper of any cultural factors included.
Vargas-Porras and colleagues (43)	The authors adopted Mercer’s ‘Becoming a Mother Theory’ (53) and evaluated the effect of the intervention on measures of becoming a mother, functional social support, mother–infant bond, and perceived maternal self-efficacy.	Some sessions occurred in the home and also via the telephone. The first visit occurred within 6–10 days (sessions alternated between home visits and phone calls), with the last visit occurring at 4 months after birth. The number of sessions offered: 8 (*4X home-based & 4X telephone*). Length of session: alternating between 15 to 90 min long One-to-one sessions	The Maternal Attachment Inventory (MAI) was developed by Müller (52).	No mention in the paper of any cultural factors included.
Chung and colleagues (44)	The education sessions used CDs and manuals and examples of topics included: (1) bathing the baby, umbilical cord care and expressing breastmilk; (2) breastfeeding challenges; (3) baby’s diet; (4) ‘Tips on caring for your baby’ on infant behaviours, and communication cues, along with a handout on play practices.	Sessions occurred in the postnatal nursing centre and/or in the home. Number of sessions offered: 5 Length of session: 40 min One-to-one sessions	Used observation with a researcher reviewing the video recording of the mother-infant interactions using the Nursing Child Assessment Teaching Scale (NCATS) developed by Sumner (55) to score mother–infant interactions. The Chinese version of the Parenting Sense of Competence (C-PSOC) Scale was developed by Ngai and colleagues (56).	No mention in the paper of any cultural factors included. The author did mention the Taiwanese ritual of ‘Do the months’ (30-day period). Also, some results may have been influenced by Taiwanese cultural factors such as modesty and humility.
Sanders and Buckner (41)	Administration of the NBO (54)	The author did not describe where the intervention took place. Number of sessions offered: 1 Length of session: 20–30 min One to one	NBO Parent Questionnaire (41)	No mention in the paper of any cultural factors included. The author concluded that further studies should be conducted to determine if the NBO could be used in various cultures.
Hoifodt and colleagues (32)	Administration of the NBO (54)	The sessions occurred in the maternity ward, during a home visit and then at a well-baby clinic 4 weeks after birth. Number of sessions offered: 1 to 3 Length of session: 15 to 40 min One to one	Maternal Antenatal Attachment Scale (MAAS) (60)	No mention in the paper of any cultural factors included.
Cheetham and Hanssen (34)	Administration of the NBO (54)	The session occurred in the maternity ward on day 2 postnatal. The home-based interview occurred 2 weeks after discharge. Number of sessions offered: 1 Length of session: 45 min One to one	None as the study was qualitative.	No mention in the paper of any cultural factors included.

The outcomes from the educational intervention that commenced during pregnancy appear to support the development of the maternal-fetal relationship. Üstüner and colleagues [31] reported that the post-test mean MAI score was higher than the pre-test, with the mean score of the intervention group significantly higher. The findings suggest that health counselling and follow-up care provided by midwives and nurses contributed significantly to enhancing and strengthening the mother-infant relationship. While Razurel and colleagues [42] reported that it was the mothers who received a postnatal interview that experienced a significant improvement in the mother–child relationship and experienced a greater sense of parental self-efficacy. These findings suggest that standardized pre and postnatal psycho-educational interviews positively supported the mother-infant relationship. The outcomes from the parenting education interventions that provided both general and targeted education also appeared to support the development of the mother-infant relationship. Vargas-Porras and colleagues [43] identified that the intervention group experienced a stronger mother-infant bond than the control group. Interestingly, the control group scores for the mother–infant bond showed a decrease between the first and the final measurement. The intervention group at four months postnatal showed efficacy in improving the process of becoming a mother. The findings concluded that the multimodal nursing intervention for first-time mothers was more effective when compared to standard care at promoting social support, mother–infant bond, and perceived maternal self-efficacy. The authors proposed that the high scores experienced in the intervention group in becoming a mother and the mother–infant bond were consistent with Mercer’s theoretical framework that both becoming a mother and maternal identity develop simultaneously with the mother-infant bond [43]. Likewise, Chung and colleagues [44] identified that postnatal education improved the overall mother–infant interaction quality in the intervention group during the 6 months after birth, although, there was no effect on the response to the ‘distress’ aspect of that interaction. Postnatal education that focused on infants’ states, behaviours, and communication cues did not appear to improve C-PSOC as there were similar results between the groups concerning parenting competence. Additionally, there were lower C-PSOC scores in both groups in the first month, after which scores increased until the third month after birth. The findings support that the postnatal education was helpful and improved the quality of mother–infant interactions for first-time mothers. Specifically, regarding the infants’ abilities and how to play with their infant during the first 6 months after birth [44].

There were three studies [32, 34, 41] that specifically targeted education on enhancing the relationship using the NBO. Sanders and Buckner [41] found no significant barriers to implementing the NBO although, there were mixed responses from the nurses on whether they could include the NBO session in their routine care. The participant mothers rated the increase in knowledge of their infants and how to respond and interact as high, stating that they found it useful, and it encouraged them to experience their infant. Hoifodt and colleagues [32] reported that the experimental group experienced significantly higher benefits from the postnatal follow-up compared to the control group. In particular, they reported learning more about their baby’s signals and needs with sleep/sleep patterns, social interaction and crying/fuzziness. Interestingly, it appeared that the benefits of the NBO were limited for the self-reported mother-infant relationship. The results suggest that the benefits of the NBO may be limited within a general population sample of participants that are well-functioning. Cheetham and Hanssen [34] reported the NBO helped mothers gain a new understanding of the baby’s communication, specifically it encouraged the mothers to interact and play or talk with their baby more. The NBO also assisted the mothers to know how to develop positive mother-infant interaction which impacted confidence in their parenting abilities. Mothers also found important aspects such as communication and techniques to soothe and calm their baby, positively impacted their competency and confidence. The findings from these studies suggest that the NBO could be a feasible intervention that may enhance engagement for some mothers and may assist in fostering a positive mother-infant relationship.

## Discussion

This scoping review identified interventions provided by a midwife or nurse working in a maternity setting that appeared to support the development of the maternal-fetal or mother-infant relationship amongst a low-risk population of women from pregnancy, and up to six weeks postnatal. This review also explored the types of interventions, including the format and delivery, and if any outcomes had been measured. Additionally, the interventions were examined to identify if consideration of cultural factors had been applied.

A benefit to the mother-infant relationship was seen during pregnancy where the mother was encouraged to view the fetus as separate from herself through abdominal palpation or by perceiving movements. Pregnant women who count movements are more likely to communicate with their baby, by talking and caressing their abdomen and also thinking about their baby’s resemblance to themselves and also to their family [46, 47]. Following birth, teaching mothers’ activities, specifically where they engaged in singing, massage, or skin-to-skin, as well as targeted educational pre and postnatal sessions also appeared to benefit the relationship. Mercer and Walker [48] theorised that the most effective way to enhance mother–infant interactions and maternal knowledge is through providing interactive reciprocal interventions.

Notably, the majority of studies incorporated an educational approach, either one-on-one or in a group setting, and used a combination of techniques such as demonstration, lectures, and additional resources. Teaching was mainly focused on increasing the woman’s knowledge of prenatal or postnatal topics and learning activities [29, 31, 33, 35, 37, 39, 43, 44], which specifically targeted improving the mother-infant relationships [30, 32, 34, 36, 41, 42]. Educational programs appear to be useful to improve knowledge and understanding of mother-infant interactions. Mothers have commonly reported feeling inadequately prepared for the role and have expressed the need for education and support interventions to help during the transition to the mothering role [43]. This is particularly important for first-time mothers who may lack experience in their new role. This lack of experience and knowledge may increase anxiety and impede confidence, negatively impacting the adaption to the maternal role [44]. Adapting successfully to motherhood influences the mother’s ability to nurture her infant and meet their physical, emotional, behavioural, and social needs [49]. If maternal confidence is compromised during the transition, the emerging mother-infant relationship may be disrupted with the mother unable to appropriately respond to their infant’s cues [49].

Gao and colleagues [50] suggested that interventions that occur consecutively in the prenatal and postnatal periods might be more successful. Additionally, Çinar and Öztürk [51] suggest that midwives and nurses were well placed to provide educational support during the postnatal period. All studies included in this review described interventions provided by the midwife or a nurse. Most commonly education in the pre-natal period was provided in a group setting [31, 35, 36, 39], with only one study offering one-on-one sessions [42]. In contrast, postnatal education was mostly delivered using a one-to-one approach [30, 32, 34, 41, 42, 44], with only one study adopting a group setting [33], and only one study offering a combination of individual and group sessions [43]. It appears that prenatal interventions are more likely to take place in a group setting, whereas postnatal interventions are more likely to be offered in one-on-one settings. The number and length of sessions varied significantly, with a majority of the prenatal [29, 35–37, 39] and postnatal [30, 33, 43, 44] interventions having been offered on more than one occasion. Despite there being no similarities in the number of sessions and the length of the interventions, it appears that providing educative support can assist in the development of the mother-infant relationship.

This review found that the maternal-fetal [29, 31, 35–37, 39], and/or the mother-infant relationship [30, 32–34, 41, 43, 44], was strengthened proceeding a specific activity or educational sessions. This was commonly explored through self-reported pre to post-measures of attachment. It is noteworthy, that most studies used self-reported validated tools to measure either maternal-fetal or mother-infant bonding and/or attachment, with the PAI [52] being the most popular tool [29, 31, 35–37]. Additionally, the MAI [53] was another tool that was used frequently [30, 31, 43].

Additionally, this review explored if there were any cultural considerations included in any of the studies. Although there were a variety of countries that explored interventions to strengthen the mother-infant relationship, the majority of studies did not describe any consideration of cultural factors. Only one study explored bonding from a cultural perspective, integrating western ideas with Korean traditional practices [39]. Interestingly, Celik and Ergin [29], Üstüner and colleagues [31] and Sanders and Buckner [41], recognised that there was insufficient diversity among participants, calling for further research to address this gap.

### Strengths and limitations

The strengths of this scoping review include a rigorous search strategy accessing a wide range of databases and the adherence to the JBI scoping review guidelines and the PRISMA-ScR checklist [24, 25]. The limitations included only reviewing full texts written in English, which may have limited the number of culturally focused studies. Additionally, there was a diverse range of study designs, the timing of the intervention and outcomes measured, with a variety of self-reported tools used to measure bonding and/or attachment. This heterogeneity in approach limits the ability to generalise the findings from this review. Finally, this scoping review did not include a quality assessment of articles.

## Conclusion

This scoping review has mapped a variety of interventions undertaken by midwives or nurses which appear to benefit the maternal-fetal and/or mother-infant relationship. Although the delivery and format of the interventions varied, it appears that providing focused activities and targeted education during the pre and postnatal periods support the development of the mother-infant relationship. Midwives are well placed to play a key role in this. Significantly, there was insufficient research that considered the influence of culture in supporting the mother-infant relationship. Further research is required to adequately understand and develop interventions that are culturally appropriate and can be integrated into care provided by midwives.

## Electronic supplementary material

Below is the link to the electronic supplementary material.

Supplementary Material 1

Supplementary Material 2

## Data Availability

All data analysed during this study are included in this published article [and its supplementary information files].

## List of Abbreviations

C-PSOC: Chinese version of the Parenting Sense of Competence
MAI: Maternal Attachment Inventory
MAS: Maternal Attachment Scale
MFAS: Maternal-Fetal Attachment Scale
MIBS: Mother-To-Infant Bonding Scale
NBO: Newborn Behavioral Observations
NCATS: Nursing Child Assessment Teaching Scale
PAI: Prenatal Attachment Inventory
PAI-R: Prenatal Attachment Inventory – Revised
PEPI: pre, and postnatal psycho-educational interviews
PEC: Prenatal education course
PRISMA-ScR: Preferred Reporting Items for Systematic reviews and Meta-Analyses extension for Scoping Reviews
WCC: Well Child Clinic
