# Supplementary material for: The role of midwives in supporting the development of the mother-infant relationship: a scoping review
Source: BMC Psychol. 2023 Mar 14;11:71. doi: 10.1186/s40359-023-01092-8 (PMC10015829; doi:10.1186/s40359-023-01092-8)
Supplement: Supplementary file 1 — Supplementary Material 1 [file 40359_2023_1092_MOESM1_ESM.docx]

## Data extraction form for scoping reviews

| Title, Author, journal | Year of publication | Population | Concept | Context | Methodology | Outcome/Key Findings |
| --- | --- | --- | --- | --- | --- | --- |
| Bellieni, C. V.; Ceccarelli, D.; Rossi, F.; Buonocore, G.; Maffei, M.; Perrone, S.; Petraglia, F  Is prenatal bonding enhanced by prenatal education courses? Minerva Ginecologica Apr 2007;59(2):125-9 | 2007 | Convenience  sampling.  Recruited in first and second trimester of pregnancy.  N=77  n=36 intervention group  n= 41 control | To assess the influence of prenatal education course (PEC) on prenatal mother-baby bonding and parameters of postnatal interaction.  *Intervention group:* received five 1-hour prenatal education course (basics of fetal physiology and development, singing sessions, dance sessions,  massage-through-the-womb sessions).  *Control group:* received the first trimester fetal echography visit and did not attend the PEC. | Italy | Quasi-randomised design | The PAI score in the intervention group was significantly higher than the control group. Furthermore, there was no significant difference between the intervention and control in the postnatal evaluation except for women in the intervention group reported higher rates of unexplained crying (P<0.05). Recognising that there was no information surrounding the process of randomization and also the reliability or validity of the population and postnatal questionnaires is a limitation. The findings suggest that prenatal education courses which are centred on communication between mother and fetus enhance prenatal attachment. The authors suggest that further research is needed to assess if the PEC promotes maternal infant attachment, as it was not specifically evaluated. |
| Celik, M.; Ergin, A.  The effect on pregnant women's prenatal attachment of a nursing practice using the first and second Leopold's maneuvers Japan Journal of Nursing Science: JJNS;17(2):e12297 | 2020 | Convenience  sampling  Pregnant  >18 years  Literate  Singleton pregnancy  No communication problems  No complex medical or psychosocial conditions.  Recruited the 28th week of pregnancy  N=100  Experimental group (n=50)  Control group (n=50) | To determine the effect on pregnant women's prenatal attachment of a nursing practice using the first and second Leopold's maneuvers.  *Intervention:* Education about fetal development & first & second Leopold's maneuvers. The Leopold's maneuvers undertaken at 28-, 32- & 36-weeks’ gestation.  *Control received:* standard care | Turkey | RCT | There was a statistically significant differences between the experimental and control groups, with the women in the experimental group recording a (p = .001, p < .01) positive effect on their prenatal attachment scores at the 32nd and 36th weeks of pregnancy compared to the control group. Recognising that while the study was only conducted at one pregnancy center in Turkey is a limitation, the findings suggest that engaging mothers in routine antenatal care practice such as Leopold's maneuverer can positively influence the developing relationship, by improving the woman’s awareness of her fetus. Additionally, sharing the findings from the abdominal examination, such as FHR with the woman may help her perceive and visualise her fetus in turn strengthening prenatal attachment. The authors suggest that this may promote maternal infant attachment, but this was not specifically evaluated. |
| Chang, S.; Park, S.; Chung, C. Effect of Taegyo-focused prenatal education on maternal-fetal attachment and self-efficacy related to childbirth Daehan Ganho Haghoeji Dec 2004;34(8):1409-15 | 2004 | Convenience  sampling.  Pregnant women (20-36 weeks of gestational age).  Singleton pregnancy  No pregnancy complications or diseases.  N=49 | To examine the effect of Taegyo-focused prenatal classes on maternal-fetal attachment and self-efficacy related to childbirth.  Taegyo classes:  Total of 8 hours (2 hours weekly for 4 weeks) Taegyo-focused prenatal education.  This included: lectures, demonstrations, practice, training, discussion, and sharing of experiences. understanding ability of fetus to respond,  sharing motivation, purpose of pregnancy, and preconceptions of experiencing childbirth, training in maternal-fetal interaction, writing letters and making a declaration of love to unborn baby. | Korean | Pre-experimental design (pre & posttest). | The MFAS score was significantly higher from pre to post-test (p<.001), indicating that the Taegyo-focused prenatal classes positively impacted maternal-fetal attachment scores, specifically, subscales ‘attributing characteristics and intentions to the fetus’ and ‘role taking’. Recognising that there is a small sample size and no control group in this study is a limitation. The findings suggest that integrating prenatal education with Traditional Korean Taegyo-focused prenatal classes can strengthen maternal-fetal attachment. |
| Cheetham, NB & Hanssen, AT "The Neonatal Behavioral Observation System: A tool to enhance the transition to motherhood", Vard i Norden, vol. 34, no. 4, pp. 48-52. | 2014 | Norwegian speaking  Primiparous  No complication  Vaginal birth.  N=4 | To create an understanding of the mothers’ experience with the NBO intervention model.  Intervention: 1 X 45-minute NBO, two days after birth. | Norway | Phenomenological qualitative study | Three themes identified interviews:   1. A new understanding of the baby 2. A feeling of greater competence and confidence 3. Being treated as an individual   The findings reported that the transition to motherhood can be positively influenced by guidance from health professionals through NBO. |
| Chung, F. F.; Wan, G. H.; Kuo, S. C.; Lin, K. C.; Liu, H. E Mother-infant interaction quality and sense of parenting competence at six months postpartum for first-time mothers in Taiwan: a multiple time series design  BMC Pregnancy & Childbirth Sep 06 2018;18(1):365 | 2018 | Convenience  sampling.  Primiparas  20–34 years old  Term  Singleton  birth  No diagnosed complications  Speaks Mandarin or Taiwanese  Lived in northern Taiwan.    N=81  Experimental group (n=41)  Control group (n=40) | How postpartum  parenting education influenced first-time mothers’ mother–infant interaction quality and parenting sense of  competence.  Experimental group: received education ‘tips on caring for your baby’ [40-minute content].  Five sessions (first week, 1^st^, 2^nd^, 3^rd^ & 6^th^ months after birth. Videotaped playing with baby then after they received general postnatal advice.  control group: did not receive any discussion of mother-infant interaction or parenting confidence. | Taiwan | Single-blind multiple time series  Design. RCT | Postnatal education appeared to improve the overall mother–infant interaction quality in the experimental group during the 6 months after birth, although, there was no effect on the response to distress aspect of that interaction. The postpartum education focusing on infants’ states, behaviours, and communication cues did not appear to improve PSOC as there were similar results between the experimental and control group in relation to parenting competence, with lower PSOC scores in both groups at the first month, after which it increased until the third month after birth. Recognising that it the participants were not randomly assign and approximately 60% of the participants remained in postpartum nursing facilities rather than in their own homes is a limitation. The findings support that the postnatal education was helpful and improved the quality of mother–infant interaction for first-time mothers specifically on the infants’ abilities and how to play with infants during the 6 months after birth. |
| Gürol, A.; Polat, S.  The effects of baby massage on attachment between mother and their infants.  Asian Nursing Research 6(1):35-41 | 2012 | Non-probability convenience sampling.  Resident of Erzurum city center  Graduated minimum secondary school  No condition preventing undertaking infant massage  No sight or hearing deficit  No previous knowledge or experience with baby massage  Primipara  Breastfeeding  Babies with the  birth weight of 2,600-4,000 g  singleton  birth between 38-42 weeks gestation  Apgar >7 at 1 & 5 minutes.  Two groups:  Experimental n=57 & control n=60 | To examine the effect of baby massage  on the mother-infant attachment.  Experimental group: Received three home visits where education provided on baby massage. Home visits occurred during the first week & then 15th & 38th day postpartum.  Babies received fifteen minutes of massage from their mothers each day for 38 days. Control group routine care | Turkey | Quasi-experimental | When comparing the MAI scores between the groups the intervention group maternal attachment score had increased post-test and was significantly higher (p <.001) compared to the control. Although the control group did experience an increase in maternal attachment score between pre and post-test, it was considerably lower than the intervention group. Recognising that the sample size was small, and the participants were not randomised but self-selected is a limitation. The findings suggest that baby massage is an effective technique to strengthen the mother-infant attachment. |
| Helk Souza, L.; Sperli Geraldes Soler, Z. A.; Sperli Geraldes Santos, M. L.; Sperli Geraldes Marin Dos Santos Sasaki, N.  Investigacion y Educacion en Enfermeria;35(3):364-371 | 2017 | Convenience  Sampling  Postnatal women day 1 & 10 postnatal  Literate  No physical or cognitive limitation.  N=200 | To characterize the demographic  profile of puerperae and variables regarding  delivery and to analyze the degree of the bond between puerpera and child, both in isolation and associated with experiences during and after delivery.  Interviews, two instruments:  Instrument 1 – sociodemographic information type of delivery, pain during  delivery, and skin-to-skin contact. Instrument 2 - Mother-to-Infant  Bonding Scale (MIBS). | Brazil | Cross-sectional study | The maternal-infant bond was not significantly influenced by type of delivery or pain during birth. It was, however, the lack of early skin-to-skin contact between mother and baby that negatively influenced bonding. Those women who did not have contact with their newborns showed significantly more sadness than those women who had skin-to-skin contact. Recognising this that some of the participants declined to answer the questions which could impact on the results, also some data was not evaluated as it was deemed irrelevant. The author also experienced difficulties interviewing participants who had health insurance. The findings suggest that early skin to skin contact is crucial to supporting the developing mother-infant relationship. |
| Hoifodt, R. S.; Nordahl, D.; Landsem, I. P.; Csifcsak, G.; Bohne, A.; Pfuhl, G.; Rognmo, K.; Braarud, H. C.; Goksoyr, A.; Moe, V.; Slinning, K.; Wang, C. E. A.Newborn Behavioral Observation, maternal stress, depressive symptoms and the mother-infant relationship: results from the Northern Babies Longitudinal Study (NorBaby) BMC Psychiatry 06 15 2020;20(1):300 | 2020 | Convenience  sampling.  Norwegian speaking  Pregnant  N=196  Intervention group n= 82  Comparison group: n=114 | To evaluate the NBO as a universal preventive intervention within the regular well-baby  clinic service by investigating the association between receiving the NBO and measures of depressive symptoms/  parental stress and the mother-infant relationship in the  first 4 months postpartum.  Comparison group: routine care.  Intervention group: received between 1- 3 NBO sessions, duration of 15 to 40 mins. These sessions occurred: in the maternity ward (within 2 days of birth); at home 7-10 days and at the well-baby clinic 4 weeks postnatal. | Norway | Longitudinal study non-randomised cluster-controlled design | The NBO did not appear to show a significant benefit to the enhancing the mother-infant relationship. Interestingly, the experimental group reported significantly higher benefit of the postpartum follow-up compared to the control group. In particular, they reported learning more about their baby’s signals and needs in relation to sleep/sleep patterns, social interaction and crying/fuzziness. Also, it appeared that the NBO was limited for participants with depressive symptoms and parental stress, as there was no association of lower of depressive symptoms and parenting stress in the experimental group. The results suggest that the benefits of the NBO may be limited within a general population sample of participants that are well-functioning. Recognising that not all participants in the experimental group received the 3-session intervention, with 15% not receiving any sessions. Also, the experimental group received one additional follow-up session compared to the control which may have contributed to uncertainty in the results is a limitation. The findings suggest that the participants who received the NBO learned more about reading their infant’s signals in everyday situation. |
| Malm, M. C.; Hildingsson, I.; Rubertsson, C.; Radestad, I.; Lindgren, H.Prenatal attachment and its association with foetal movement during pregnancy - A population based survey Women & Birth: Journal of the Australian College of Midwives Dec 2016;29(6):482-486 | 2016 | Pregnant women: 34-42 weeks gestation  Speak Swedish language  Singleton pregnancy  N=456 | To investigate the association between the magnitude of foetal movements and level of  prenatal attachment within a 24 h period among women in the third trimester of pregnancy.  All women were invited to complete two questionnaires. | Sweden | Prospective population-based survey | The results from the PAI-R indicated significantly higher scores of attachment for the women who perceived frequent fetal movements on several occasions within a 24-hour period. Higher scores in all subscales on ‘anticipation’, ‘differentiation’ and ‘interaction’ occurred when women perceived many fetal movements on numerous occasions. Recognising that the questionnaire developed to identify the magnitude of fetal movements has not undergone reliability and validity testing is a limitation. The author suggests that these findings may have occurred because the frequent movement felt facilitated a greater sense of attachment in the women alternatively, the woman may have had a strong sense of prenatal attachment therefore this influenced their sensitivity to the baby’s activity. These results suggest that the magnitude of foetal movements felt along with the perception of frequent foetal movements in late pregnancy was strongly associated with prenatal attachment. Therefore, engaging women during antenatal care to recognise foetal movements and sleep-wake cycles may strengthen their prenatal attachment. |
| Midtsund, A.; Litland, A.; Hjalmhult, E.  Mothers' experiences learning and performing infant massage-A qualitative study  Journal of Clinical Nursing ;28(3-4):489-498 | 2019 | Convenience  sampling.  >18 years  Norwegian language.  N=12 | To explore the experience of learning infant massage among  mothers who are having insecurity and stress in their transition to motherhood.  Infant massage.  Mamma Mia Six-week program with weekly sessions. Each session involves course on infant massage and education on topics such as interaction, attachment and children’s behaviour. | Norway | Qualitative. Explorative design | The author identified one main theme ‘a relief with an opportunity for emotional and physical connection with the baby’, with four categories “appreciating the structure,” “providing self-esteem and self-confidence,’ ‘connecting with the baby’ and ‘discovering the baby's presence’. The mothers reported that it was a positive experience and helped them feel attached to their baby during the infant massage and found that they integrated this knowledge into their everyday life. It helped establish a deeper connection with their baby and offered an opportunity to spend time with them. They also appreciated the baby’s response to massage and found that they felt energised. Infant massage also provided the women with a useful technique to calm their baby down without breastfeeding, with the women reporting that they felt empowered to use what they had learnt. The women also valued the education that they received surrounding baby cues, signs and behaviours. Recognising the small sample size and that the interviews were conducted from a few weeks to six months after participation in the program, with memory changing over time it therefore this may have influenced their answers. The findings suggest that baby massage is an effective technique to strengthen the mother-infant attachment. |
| Nishikawa, M.; Sakakibara, H.Effect of nursing intervention program using abdominal palpation of Leopold's maneuvers on maternal-fetal attachment Reproductive Health 2013;10 (1) (no pagination)(12): | 2013 | Convenience  sampling.  Pregnant Japanese women  Singleton pregnancy  >30 weeks pregnant  Regular prenatal care through the hospital  No complications  N=227  Intervention group: n=88  Control group: n=139 | Intervention group: received education through the pre-mothers’ classes (X3; 30, 32 & 34 weeks gestation) and the abdominal palpation intervention. 1:1 with midwife explaining the fetal position then group discussion (approx. 1-1.5 hrs)  Control group: routine care receiving education through the pre-mothers’ classes (X3; 30,32 & 34 weeks gestation). | Japan | Experimental | The PAI score in the intervention group was significantly higher (P< 0.01) at the 32nd, 34th, and 36th weeks’ gestation compared to the baseline. The study demonstrated that the intervention was effective for promoting maternal-fetal attachment. In addition, maternal awareness (P < 0.01) and the frequency of talking to the fetus (P < 0.05 or P < 0.01) was higher in the intervention group than in the control group at the 32nd, 34th, and 36th gestational weeks. Recognising that the participants were only from one setting, and they were not randomly assigned is a limitation. The findings suggest that as pregnancy progresses maternal-fetal attachment develops and interventions such as abdominal palpation assist the woman with awareness of the fetal position. Awareness of the fetal position and talking to the fetus appear to be effective in supporting maternal-fetal attachment. |
| Persico, G.; Antolini, L.; Vergani, P.; Costantini, W.; Nardi, M. T.; Bellotti, L.  Maternal singing of lullabies during pregnancy and after birth: Effects on mother-infant bonding and on newborns' behaviour. Concurrent Cohort Study  Women & Birth: Journal of the Australian College of Midwives;30(4):e214-e220 | 2017 | Convenience  sampling  Pregnant women (24 weeks of gestational age)  >18 years  Italian language &  uncomplicated singleton pregnancies.  N=196  A total of twenty-eight women did not complete the study (n=14) experimental and (n=14) from the control group  n=83 singing cohort (sing  lullabies during pregnancy)  n=85 concurrent cohort (non-singing women) | To assess the effects of mothers singing lullabies on both prenatal attachment and postnatal mother–infant bonding  by means of established and validated questionnaires.  14 weekly sessions (from 24 weeks gestation).  Singing cohort: provided traditional,  loving and playful songs. Prior to the antenatal session then after four weeks invited to continue singing at home. Received additional education about maternal singing in the antenatal classes. The singing cohort asked to record frequency of singing each week & reaction of the fetus.  Concurrent cohort: received standard antenatal classes singing lullabies was not covered. Women left free to sing if they wanted to. Women were asked if they sung lullabies regularly. | Italy | Quasi-randomized | The PAI score in the intervention group and the control were not of stastical interest. The mother-infant bonding score did demonstrate a statistically significant difference in bonding at three months in the intervention group compared to the control. The study also found that women mostly chose to sing in the evening and experienced positive feeling when singing lullabies such as serenity, relaxation and harmony with the baby, along with an increased awareness of change in foetal behaviour. Most mothers in the intervention group continued singing lullabies to their baby after the birth and found that this activity enriched their relationship. Singing lullabies also appeared to have a positive impact on maternal stress (p<0.05), with women reporting that it was easier to calm their infant. Similarly, women in the intervention group reported a positive impact on newborn behaviour (p < 0.0001) with the frequency of crying episodes significantly lesser during the first month postpartum. Recognising that the participants were not blind to the two cohorts’ women in the control group (8%) although they did not receive education on singing lullabies spontaneously did sing is a limitation. The findings suggest that singing lullabies during pregnancy and continuing after birth appears to be effective in enriching the mother-infant relationship, with women highlighting the strong emotions and feeling they experienced when singing antenatally and postnatally. Furthermore, singing lullabies may also have a positive effect on newborn behaviour, with less episodes of crying and colic. |
| Razurel, C.; Antonietti, J. P.; Rulfi, F.; Pasquier, N.; Domingues-Montanari, S.; Darwiche, J.  The impact of pre- and post-natal psycho-educational intervention on the construction of parenthood Archives of Women's Mental Health;20(3):469-472 | 2017 | Convenience  sampling.  >18 years  French language  Nulliparous.  Group 1 (n = 26) received a prenatal and postnatal interview.  Group 2 (n = 23) received standard practice (prenatal  interview only). | To assess the added value of  standardized psycho-educational prenatal and postnatal  interviews on the mental health of the mother, her sense  of self-efficacy, as well as the mother-and-child relation-  ship, and the quality of the couple at 2 months (T2) and  3 months (T3) postpartum.  Group 1: prenatal interview from 30 weeks gestation and postnatal interview at 6 weeks. Interviews were 1 hour each using questionnaire-guided  manualised psycho-educational perinatal interview  (PEPI). The PEPI helped mothers; find solutions  related to their needs; decrease perceptions of  adaptation-associated stress; promote personal re-  sources and increase their awareness of self-efficacy | Switzerland | Pilot study, pre and post comparison design. | The group one who received a postnatal interview experienced a significant improvement in the mother–child relationship at T2 and T3 and also experienced a greater sense of parental self-efficacy. There were encouraging results in group one regarding a reduction in the mothers’ depression, stress, and anxiety. Recognising that the sample size and the lack of details of the randomization process is a limitation. The findings suggest that standardized pre, and postnatal psycho-educational interviews positively support the mother-infant relationship although larger sample are required to confirm these results. |
| Sanders, L. W.; Buckner, E. B.  The Newborn Behavioral Observations system as a nursing intervention to enhance engagement in first-time mothers: feasibility and desirability.  Pediatric Nursing;32(5):455-9 | 2006 | Convenience  sampling.  First-time mothers  >19 years  Baby rooming in (postnatal)  Nurses.  First time mothers:  N=10  Nurses: N=20 | X 1 Newborn Behavioral Observations (NBO) session average length 25 minutes.  Interview: after the session Desirability: what they liked about the intervention, what they learnt and how it helped with caregiving.  Newborn Behavioral Observations (NBO) Parent  Questionnaire completed after the session. Questionnaires on the feasibility and desirability of using the NBO was completed by RN. | USA | Pilot | No significant barriers to implementing the NBO as an intervention from the mothers or the investigators view, although there were mixed responses from the nurses with if they could include an NBO session in their routine patient care, (n=7) answered yes, (n=6) answered no, and (n=7) were unsure. Also, it was unclear who the responsibility of performing the NBO, with nurses answering it should be completed by the mother’s nurse (n=5), the infant’s nurse (n=8), and a specialist (n=11). The participant mothers rated the efficacy in increasing their knowledge of their infants and how to respond and interact as high, stating that they found it useful, and it encouraged them to experience their infant. Participants also rated the NBO high as an overall learning experience as it educated them on different ways they can actively participate in caregiving. Recognising that there were sample size and also insufficient diversity amongst participants is a limitation. The findings from this study suggest that the NBO could be a desirable and feasible intervention that may enhance engagement in first-time mothers. |
| Üstüner Top, F.; Yigitbas, C.; Cantürk, P.; Yüksel, F.  The effects of health counselling and follow-up care on maternal-infant attachment  Early Child Development and Care | 2020 | Pregnant women from 20-30 gestational weeks.  N=86  Experimental group (n=43)  Control group (n=43) | To determine the effectiveness of follow-up care  and health counselling provided by midwives and nurses in maternal-  infant attachment level and relationships between sociodemographic  factors.  The experimental group received four weeks education from a midwife (topics included: pregnancy and wellbeing, physiology of delivery and techniques to  cope with birth waves, Pilates exercises, hospital bag preparation, importance of breast milk and  correct breastfeeding techniques, newborn care and common problems, nursing care and family  planning methods and a preview of the delivery room).  Control group received no education. | Turkey | Quasi-experimental | The author identified that the prenatal attachment levels were similar in the two groups, with the pre-test mean PAI score high in both groups. The post-test mean MAI score was also higher in both groups, with the mean score of the experimental group significantly higher than that of the control group. includes women only from Turkey, therefore the results cannot be generalized is a limitation. The findings suggest that health counselling and follow-up care provided by midwives and nurses contributed significantly to enhancing and strengthening the mother-infant relationship. |
| Vargas-Porras, C.; Roa-Díaz, Z. M.; Hernández-Hincapié, H. G.; Ferré-Grau, C.; de Molina-Fernández, M. I. Efficacy of a multimodal nursing intervention strategy in the process of becoming a mother: A randomized controlled trial Research in Nursing and Health 2021;44(3):424-437 | 2021 | Convenience  sampling.  Residing in Bucaramanga,  Floridablanca, Girón, or Piedecuest  Speaks Spanish  > 18 years old  Postpartum  Primiparous  Healthy term infant  Partner support  Smartphone & internet access.  N=76  Experimental group (n=38)  Control group (n=38) | To assess the efficacy of a new  multimodal nursing intervention focused on supporting the process  of becoming a mother in first‐time mothers of term infants, through  evaluating the effect of the intervention on measures of becoming a  mother, functional social support, mother–infant bond, and perceived maternal self‐efficacy  Intervention group: received multimodal intervention “Maternal Support for Becoming a First‐time Mother”. Involved 8 sessions (4X home-based & 4X telephone). Home-based visits 90 minutes; phone 15 minutes duration. First visit occurred within 6-10 days (sessions alternated between home visits and phone calls), with the last visit occurring at 4 months after birth.  Control group: received usual postnatal care. | Colombia | Parallel‐group, double‐blind, randomized, controlled trial. | The intervention group experienced a stronger bond between mothers and their infants than the women in the control group, with both groups maintaining a high score during the 4 months. Interestingly, the control group scores for the mother–infant bond showed a decrease between the first and the final measurement. The intervention group at four months postpartum showed efficacy in improving the process of becoming a mother. Recognising that it is unclear if the content or the contact explain the results as they were not tested separately is a limitation. The findings support that the multimodal nursing intervention for first‐time mothers was more effective when compared to standard care at supporting social support, mother–infant bond, and perceived maternal self-efficacy. The author suggests that the high scores experienced in the intervention group in becoming a mother the mother–infant bond is also consistent with Mercers theoretical framework that both becoming a mother and maternal identity develop simultaneously with the mother-infant bond. |
