# Supplementary material for: The role of midwives in supporting the development of the mother-infant relationship: a scoping review
Source: BMC Psychol. 2023 Mar 14;11:71. doi: 10.1186/s40359-023-01092-8 (PMC10015829; doi:10.1186/s40359-023-01092-8)
Supplement: Supplementary file 2 — Supplementary Material 2 [file 40359_2023_1092_MOESM2_ESM.docx]

# Appendix 1. Medline search

Database(s): Ovid MEDLINE(R) ALL 1946 to July 08, 2021
Search Strategy:

| **#** | **Searches** | **Results** |
| --- | --- | --- |
| 1 | exp Mother-Child Relations/ | 21823 |
| 2 | ((mother infant or mother child or mother-to-infant or maternal-infant or maternal or maternofoetal or maternofetal or foetomaternal or fetomaternal or materno foetal or materno fetal) adj5 (attach* or bond* or relation* or interact*)).tw,kf. | 18098 |
| 3 | 1 or 2 | 35510 |
| 4 | Pregnancy/ | 909549 |
| 5 | Fetus/ | 79593 |
| 6 | Infant, Newborn/ | 625648 |
| 7 | Postpartum Period/ | 27167 |
| 8 | (pregnan* or antenat* or prenat* or foetus or fetus or foetal or fetal or newborn* or infant* or neonat* or perinatal or puerperium or postpartum or post partum or postnatal or after birth or puerperal or early child* develop* or newly born).tw,kf. | 1496585 |
| 9 | 4 or 5 or 6 or 7 or 8 | 2019962 |
| 10 | 3 and 9 | 21063 |
| 11 | Mothers/ | 47148 |
| 12 | (mother* or human females).tw,kf. | 234436 |
| 13 | 11 or 12 | 245972 |
| 14 | Object Attachment/ | 13815 |
| 15 | ((attachment* or bond* or relation* or interaction* or behav*) adj5 (emotion* or psycholog* or symbio*)).tw,kf. | 65467 |
| 16 | 14 or 15 | 77906 |
| 17 | 9 and 13 and 16 | 3358 |
| 18 | 10 or 17 | 22354 |
| 19 | exp Nurses/ | 91229 |
| 20 | exp Nursing/ | 256784 |
| 21 | exp Midwifery/ | 19820 |
| 22 | (nurs* or midwi*).tw,kf. | 502328 |
| 23 | 19 or 20 or 21 or 22 | 638836 |
| 24 | 18 and 23 | 2208 |
| 25 | exp Patient Education as Topic/ | 87323 |
| 26 | Health education/ | 61958 |
| 27 | Health promotion/ | 76834 |
| 28 | Program evaluation/ | 65198 |
| 29 | Psychosocial Intervention/ | 236 |
| 30 | Behavior Observation Techniques/ | 425 |
| 31 | (behav* observat* techn* or antenat* or prenat* or educat* or class* or promot* or campaign* or program* or pilot or project* or evaluat* or intervent* or seminar* or webinar* or tool* or train* or therap* or activit* or prevent*).tw,kf. | 12962029 |
| 32 | 25 or 26 or 27 or 28 or 29 or 30 or 31 | 13032845 |
| 33 | 24 and 32 | 1295 |
